# Examination of A Novel Nanocompositer’s Bioactivity for Use in Dentistry: Use of a Novel Nanocompositer's Bioactivity in Dentistry

**DOI:** 10.31661/gmj.v13iSP1.3663

**Published:** 2024-12-31

**Authors:** Sarvenaz Amanolahi Baharvand, Pardis Chaboki, Fateme Pourhatami, Mohamad Milad Shirvandehi, Reza Yusofvand, Payam Ali –Khiavi, Mohammad Shabestari Khiabani, Khadije Yousefi, Paria Ganji Nataj, Paria Arjmandi Sarvestani, Sepideh Karkonshayan, Reza Akhavan-Sigari

**Affiliations:** ^1^ Faculty of Pharmacy, Mazandaran University of Medical Sciences, Mazandaran, Iran; ^2^ School of Medicine, Shiraz University of Medical Sciences, Shiraz, Iran; ^3^ Department of Basic Science, Faculty of Pharmacy, Pharmaceutical Sciences Branch, Islamic Azad University, Tehrani, Iran; ^4^ Department of Microbiology, Faculty of Medicine, Arak University of Medical Sciences, Arak, Iran; ^5^ Department of Exceptional Talents, Faculty of Medicine Sciences, Lorestan University of Medical Sciences, Khorramabad, Iran; ^6^ School of Medicine, Tabriz University of Medical Sciences, Tabriz, Iran; ^7^ Dentistry faculty, Tabriz University of Medical Sciences, Tabriz, Iran; ^8^ Department of Materials Science and Engineering, School of Engineering, Yasouj University, Yasouj, Iran; ^9^ Dalian Medical University, Dalian, China; ^10^ Department of Biomedical Engineering, Faculty of Engineering, Zand Institute of Higher Education, Shiraz, Iran; ^11^ Student Research Committee, School of Medicine, Gonabad University of Medical Sciences, Gonabad, Iran; ^12^ Dreifaltigkeits-Hospital Lippstadt, Teaching Hospital of the University of Münster, Germany; ^13^ Department of Health Care Management and Clinical Research, Collegium Humanum Warsaw Management University Warsaw, Poland

**Keywords:** Nanocomposite; Nano Fast Cement, Setting Time, Nano Hydroxyapatite, Bioactivity

## Abstract

The extended setting time of mineral trioxide aggregate (MTA) is one of its primary drawbacks. An alternative to MTA, Nano Fast Cement (NFC) is a novel nanocomposite with a quick setting time. In order to develop an exceptional dental filler, hydroxyapatite nanoparticles (NHA) were introduced to NFC to examine its impact on setting time, biocompatibility, and bioactivity qualities. X-ray diffraction, scanning electron microscopy, Gilmore needle, and MTT assay were used to evaluate the specimens with 0, 10, and 20 W% hydroxyapatite. The antibacterial evaluation was conducted using Enterococcus faecalis (PTCC 1394). Using phase and microstructural studies, the production of hydroxyapatite was discovered. Both 10 and 20 weight percent prime apatite enhanced the specimens’ bioactivity and markedly decreased their toxicity behavior. More HA content resulted in greater bioactivity enhancement and toxicity decrease. These results suggest that the physical properties of NFC were not negatively impacted by the addition of NHA. In vitro, they enhanced the filler’s bioactivity.

## Introduction

**Figure-1:**
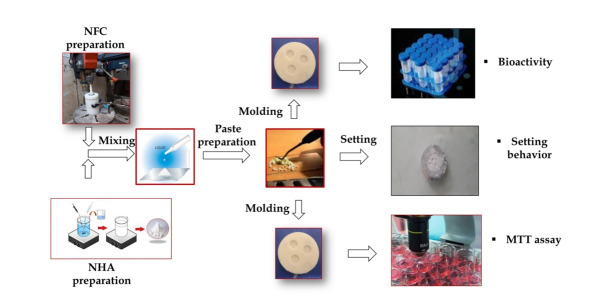
Figure 1. Material processing flow chart

**Table:** Table1. The Different Compositions of NFC/NHA Nanocomposite

**Materials**	**Powder composition **	**Hydration agent **
NFCH0	100% NFC	water
NFCH10	90% NFC + 10%NHA	water
NFCH20	80% NFC + 20%NHA	water

In dental research, calcium silicate cements (CSCs), akin to mineral trioxide aggregate (MTA) [1], serve to mend deficiencies in tooth root sides and to complete root ends after procedures [2]. MTA was initially developed as a root-end filling material due to its special therapeutic properties such as bioactivity [3], biocompatibility [4], and sealing ability [5]. Nonetheless [6], the challenges associated with it, including difficulty in handling, weakness, extended setting duration, color changes, and high expense [7], hinder its effectiveness for repair tasks [8][9]. Ideally, a dental filler material should set quickly to prevent saliva from washing it away and reduce the risk of irritating oral tissues [5]. Prolonged setting time of MTA has been shown to cause various issues with dental treatment process management [10]. The normal setting time for MTA exceeds 2.5 to 3 hours under regular conditions [14], Occasionally, achieving the final filling or cover for a tooth requires multiple attempts [11]. This shows that we need to create a new material made from calcium silicate and cement to make it set faster [12][13]. New CSCs like Nano Fast Cement (NFC) from Sanat Avaran Vista, Iran [15], have been developed with an initial setting time of 5 minutes [16][17]. NFC is a fast-setting CSC composed of zirconia oxide, silicon dioxide, and calcium dioxide [18]. In a recent laboratory experiment, NFC demonstrated a marginally shorter setting time at both the start and finish compared to Angelus MTA and Calcium-enriched cement (CEM). On the other hand, using calcium phosphate (CaP) as a material for bone grafts has had good results. When introduced into bone defects, calcium phosphates, including hydroxyapatite and beta-tricalcium phosphate (beta-TCP) [19], are safe for bodily use, stimulate bone formation, and facilitate healing [20].

Hydroxyapatite can serve as a scaffold for new bone formation and can enhance capillary [21], perivascular [22], and osteogenic cell growth in the recipient area [23]. An essential quality of retrofilling cement is its bioactivity [13], which enables the creation of a solid attachment to living tissue by producing a layer of hydroxyapatite [24][25][26][27]. However, the fundamental requirement for a material implanted in the human body is its biocompatibility [28]. The addition of hydroxyapatite to root canal sealers, dental materials, and tooth glues serves to reinforce them and enhance their effectiveness and biological response [29][30]. NHA is a potential addition with attributes such as biocompatibility, bioactivity, and osteoconductivity [31][32], making it a promising additive that could enhance the properties of NFC. This study seeks to determine the effects of integrating NHA with NFC in order to improve its biocompatibility, bioactivity, and antibacterial functions.

## Materials and Methods

**Figure-2:**
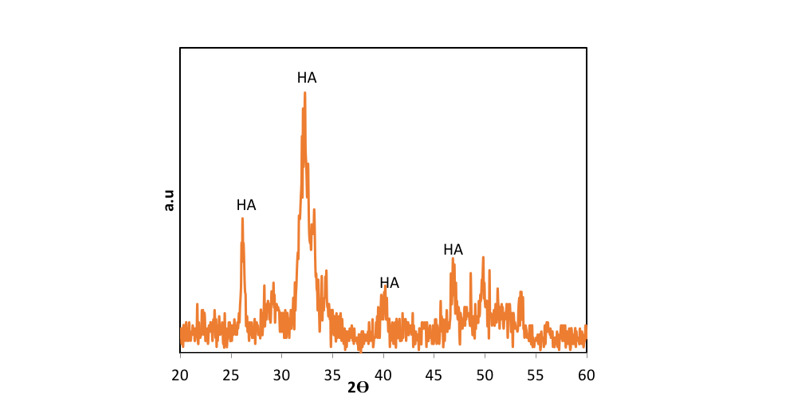
Figure 2. XRD patterns of NHA powder

**Figure-3:**
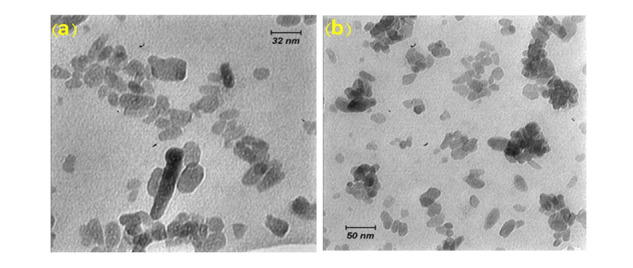
Figure 3. TEM images of NHA powders a) 32 nm resolution b) 50 nm resolution

**Figure-4:**
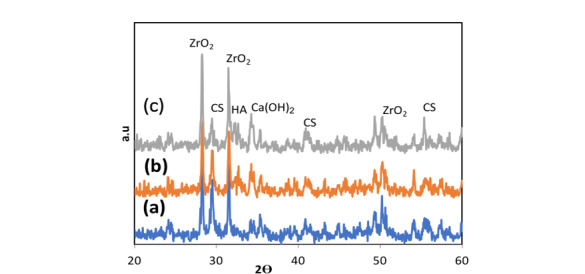
Figure 4. XRD patterns of NFCH0, NFCH10, and NFCH20 before soaking in SBF

**Figure-5:**
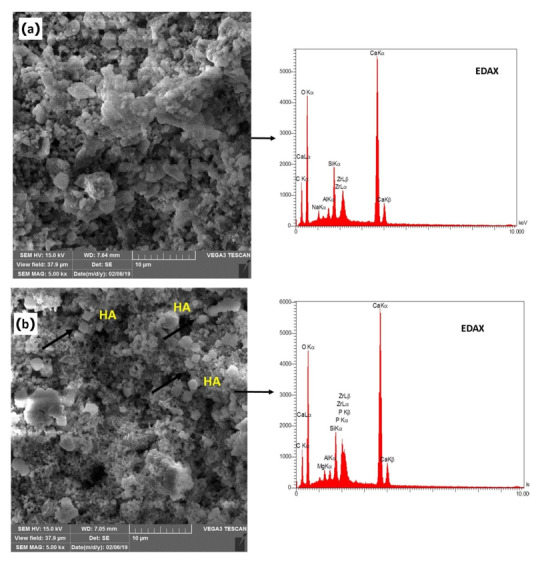
Figure 5. SEM & EDAX of NFCH0 and NFCH20 the distribution of NHA particles present in NFC

**Table:** Table2. Ion Concentrations (mM) in Artificial Body Fluid Versus Blood Plasma Levels [22]

**Formulation**	**Na ^+^**	**K ^+^**	**Ca ^2+^**	**Mg2 ^+^**	**Cl ^-^**	**HCO ^3-^**	**HPO _4_^2-^**	**SO _4_^2-^**
Human blood plasma	142	5	2.5	1.5	103	27	1	0.5
SBF	142	5	2.5	1.5	147.8	4.2	1	0.5

**Table:** Table3. XRD Peaks of Ingredients of Cement

Compound	**1th** **peak**	**2th** **peak**	**3th** **peak**	**4th** **peak**	**5th** **peak**	**6th** **peak**
**C_3_S **	29.51°	32.70°	34.41°	41.48°	51.70°	
**C_2_S **	31.98°	32.37°	40.55°			
**Ca(OH) _2_**	34.1°	47.63°				
**Zirconium oxide**	27.37°	33.40°	49.3°,50			
**C-S-H**	26.59°	29.36°	30.92°	47.73°	50.52°	
**Hydroxyapatite**	25.88°	31.77°	32.90°	39.79°	46.69°	49.49°

### Powder Preparation

The study utilized NFC as the main component, NHA as the bioactive element, and distilled water as the agent for hydration. The NHA&NFC powders were produced and characterized as detailed in previous studies [33][34]. In brief, NHA was created through the blending of calcium nitrate tetrahydrate and phosphoric acid.Calcium nitrate tetrahydrate obtained from Sigma-Aldrich, USA, exhibited a purity of 99%.The phosphoric acid, which originated from Sigma-Aldrich, USA, exhibited a purity of 85%. The nano fact cement (Sanat Avaran Vista Co., Iran) with a particle size of 3453nm was reduced to 300nm through Wet-Stirred-Media-Milling over 15 hours. The composite NFC/NHA powder was created by blending NFC and NHA with 0, 10, and 20% of NHA powder for NFCH0, NFCH10, and NFCH20, respectively.

### Paste Preparation

During this stage, the powders of the nanocomposite were mixed together with distilled water. Following this, the mixture was delicately stirred with a spatula for 30 seconds until a uniform and workable paste formed, which was then poured into a silicone mold. To identify the most effective composition for the produced calcium silicate nanocomposite, various compositions of the nanocomposite were prepared, as outlined in Table-1. The process for handling the materials is illustrated in Figure-1.

### Phase Evaluation and Microstructural Characterization

The D-50 diffractometer Siemens model was used to determine the X-ray diffraction patterns, employing Cu-ka radiation with a 1.5 A wavelength in the 2θ range of 20°- 60°. By employing JCPDS reference cards and Xpert high score software, we detected various patterns. A TESCAN-Vega 3 scanning electron microscope (SEM) was utilized to analyze the surface characteristics of samples before and after being submerged in SBF. To boost the efficiency of electricity conduction, a slim coating of carbon was added to the samples. The analysis of the samples was conducted with a scanning electron microscope, operating at a voltage of 15 kV.

### Setting Time Evaluation

The measurement of the NFC’s setting times was conducted following the standards outlined in ISO 6876/2012.The experimental mixtures were combined and transferred into a circular stainless-steel mold that has a width of 5 mm and a height of 10 mm. Once mixed, the samples were positioned in an incubator maintained at 37 degrees Celsius and 95% relative humidity. A 1 mm wide flat tip was employed, and it had a weight of 400 grams. It was firmly positioned straight down onto the tested material’s surface. This repeated itself every minute. The time for final setting was logged when the needle no longer created a mark.

### Handling Properties

The samples’ handling characteristics were evaluated with a texture analyzer (CT3, Brookfield, Middleboro, USA) equipped with a 4.5 kg load sensor. Each sample was compressed using a tool measuring 5 mm in diameter and having a volume of one cubic centimeter.This was executed with a speed rate of 0.02 mm per second after mixing the sample. By measuring the force necessary to compress the material to a designated depth, we were able to evaluate how manageable it is.

### Bioactivity Evaluation

The bioactivity was assessed following the guidelines outlined in ISO 23317 [36]. A little segment, measuring 10 mm across and 2 mm long, was fabricated and then immersed in a fluid that imitates body fluids for 14 days once it had set. Simulated body fluid (SBF) is a liquid composed of particles similar to those found in human blood. It is buffered to a pH of 7.40 at physiological temperature using HEPES/NaOH [37][38]. The chemical composition of SBF can be found in Table-2.

### Statistical Analysis

In the present study, SPSS IBM subsidized software was used to analyze the data. To evaluate the results of biological experiments, the data was displayed in each group as mean ± standard deviation. A significant difference between mean groups was analyzed by one-way ANOVA analysis and then Tukey test. In all experiments, an average of 3 measurements was used for each group, and a significant level in all statistical tests (P) was considered less than 0.05.

## Results & discussion

**Figure-6:**
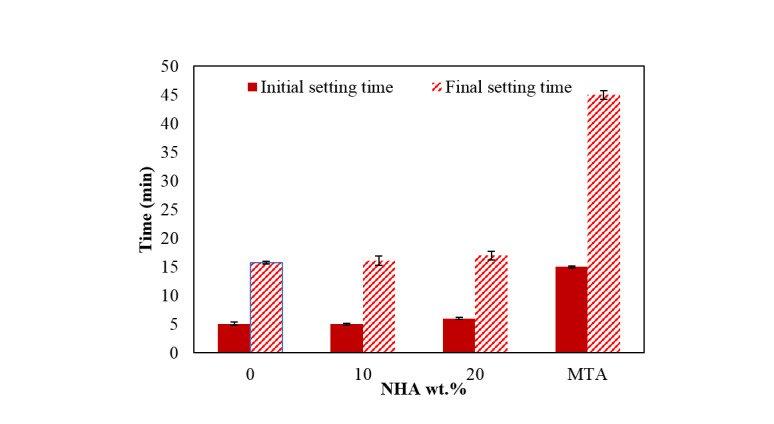
Figure 6. The effect of NHA on the rapidity of NFC initialization

**Figure-7:**
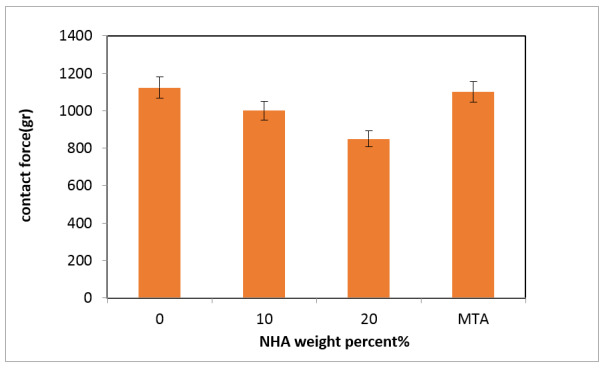
Figure 7. The effects of NHA on the handling properties of the NFC

**Figure-8:**
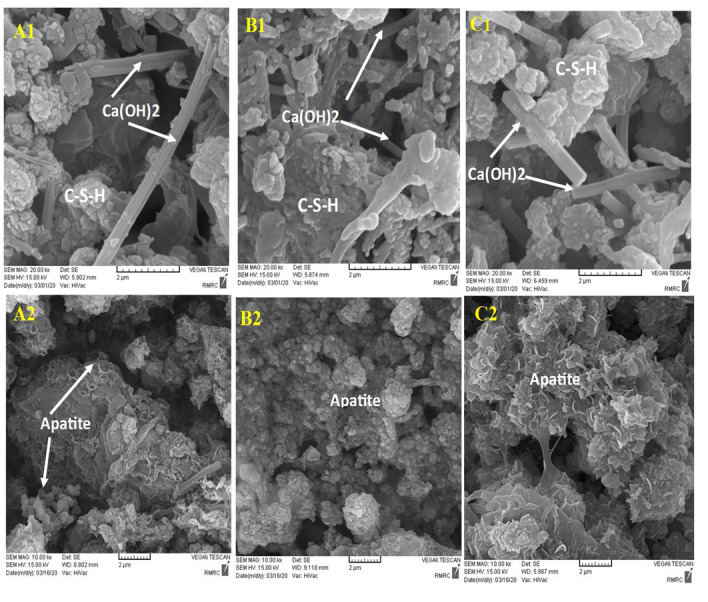
Figure 8. SEM image of the sample before soaking in SBF (A1) NFCH0, (B1) NFCH10 (C1) NFCH20 after soaking in SBF for 14 days (A2) NFCH0 (B2) NFCH10 (C3) NFC20

**Figure-9:**
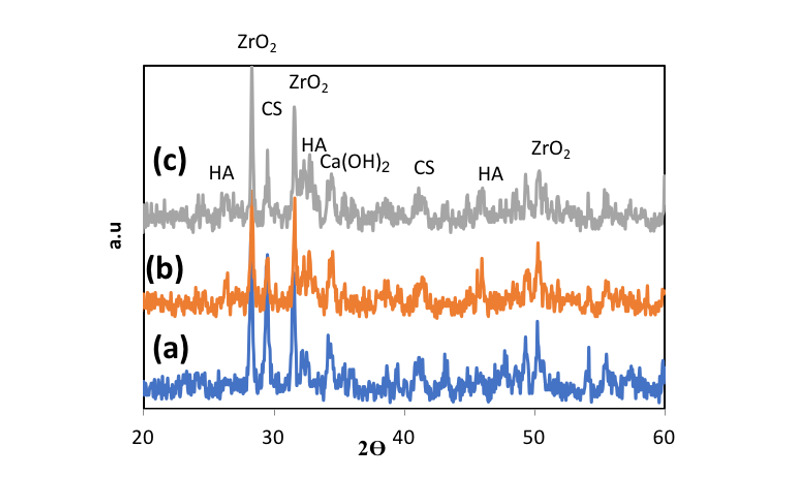
Figure 9. XRD patterns of a)NFCH0 b)NFCH10c) NFCH20 after soaking in SBF

**Figure-10:**
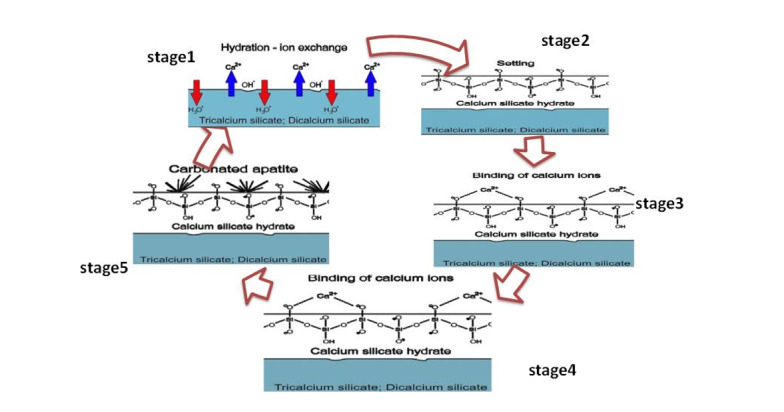
Figure 10. Schematic of the five concurrent stages of activities leading to the realization of NFC's bioactivity in vitro after the latter is immersed in virtual body fluid. ACP: amorphous phosphate calcium.

### Characterization of Synthesized Powders and Cement Pastes

In the present study, Figure-2 depicts the XRD patterns of NHA powder, exhibiting the characteristic peaks of hydroxyapatite and indicating the absence of impurities in the produced hydroxyapatite powder. Furthermore, Figure-3 displays the transmission electron microscopy images of the synthesized hydroxyapatite powder, revealing that the hydroxyapatite nanoparticles measure between 20 and 50 nm in size. This powder was utilized in the fabrication of a calcium silicate nanocomposite containing hydroxyapatite, and its impact on NFC properties was examined. Figure-4 displays the X-ray diffraction (XRD) patterns for NFCH0, NFCH10, and NFCH20.These patterns are consistent with the samples before their exposure to the simulated body fluid (SBF) solution. In Table-3, the significant peaks corresponding to the different compounds can be observed. The primary components of NFC consist of tricalcium silicate (C3S), dicalcium silicate (C2S), tricalcium aluminate (C3A), along with zirconium oxide. The addition of water results in a slight reduction of tricalcium silicate and dicalcium silicate, while it leads to the formation of calcium silicate hydrate (C-S-H) and calcium hydroxide (Ca (OH)2). The spectrum exhibits an additional peak in the NFCH10 and NFCH20 specimens at 31.7 and 32.5°, respectively. These peaks correspond to the hydroxyapatite, which was incorporated at 10 and 20 weight percent into NFC to enhance the cement’s bioactivity properties Table-3. Figure-5 displays the SEM images of the NFCH0 and NFCH20 samples, revealing fine particles dispersed on the CSH surface. Examination of the X-ray energy distribution of these particles indicates that they consist of hydroxyapatite. Calcium and phosphorus were found in the X-ray analysis of the material, attributed to the presence of apatite in the NFC.

### The Effect of Nano-hydroxyapatite on the Setting Time of NFC

The NFC consists of hydrophilic particles that solidify upon exposure to moisture [15]. This substance offers an alternative to MTA, which has a lengthy setting time that is not ideal for clinical use [39]. The setting characteristics of NFC are examined in this section, including the influence of adding NHA on the setting time. The structure and hydration of NFC result from a series of intricate reactions. The starting and finishing times for each of the different mixtures are displayed in Figure-6. The initial setting time for NFCH0 was approximately 5 minutes, with a final setting time of 15.75 minutes. MTA is a widely used dental sealer, with a setting time of 2-3 hours, The speed is considerably slow, which can result in various health concerns [40]. The findings indicate that by using pure NFC, the setting time can be reduced by 7.5-11 times compared to MTA.The rise in the amount of NHA results in a minimal change in the initial and final setting times of NFC, as depicted in Figure-6. Hydroxyapatite nanoparticles, which are calcium phosphate, do not disrupt hydration reactions and therefore have little impact on the setting time. Any slight increase in setting time may have been influenced by the powder/liquid ratio, given that samples containing NHA required a greater amount of water to achieve a suitable consistency for clinical purposes, consequently leading to an increase in the setting timeb [41][42][43][44][45][46].

### Handling Properties

The handling properties were assessed by measuring the maximum contact force needed to compress prepared pastes using a 5 mm diameter probe to a depth of 4 mm. Figure-7 compares the contact force of samples prepared with 0, 10, and 20 wt% NHA. The figure indicates that the contact force decreases for samples containing 10 and 20 wt% NHA in the NFC paste. Specifically, the contact force for the sample with 0 wt% NHA (NFC0) is 1123 gr, which decreases to 800 gr when 20 wt% NHA is used in the NFC20. The demand for advanced dental materials has led to the exploration of nanocomposites, which offer enhanced mechanical properties and bioactivity. Understanding the thermal behavior of these materials under various conditions is crucial for their application in dentistry [41][42][43][44][45][46].

### In vitro Bioactivity Test

The nanocomposite’s bioactivity was assessed through in vitro tests involving immersion in SBF at 36.5 °C for 14 days. Following this immersion, XRD and SEM analysis were conducted to examine the formation of apatite on the nanocomposite surfaces. The presence of secondary apatite on the surfaces after exposure to SBF provides insight into its bioactivity under in vitro conditions, as stated in the literature. SEM and EDAX images of three different samples before and after immersion in SBF are shown in Figure-8. The microstructure of NFCH0, NFCH10, and NFCH20 before immersion is depicted in Figure-8(A1, B1, C1), displaying a composition of CSH matrix, calcium hydroxide crystals, and some pores resulting from hydration reactions. Figure-8 (A2, B2, C2) illustrates the microstructure of NFCH0, NFCH10, and NFCH20 after 14 days of immersion in SBF, revealing a notable development of apatite on the cement surfaces. Notably, NFCH10 and NFCH20 exhibited greater homogeneity and uniformity with fewer porosities compared to the NHA-free sample (NFCH0), and the formation of hydroxyapatite crystals was more pronounced on their surfaces. Understanding the development of apatite on cement is crucial for assessing its compatibility with living tissues and its possible applications in dentistry. The XRD spectrum of hydrated samples after SBF immersion is presented in Figure-9, indicating that the spectrum of NFCH0 after soaking in SBF is similar to the spectrum of NFCH0 before soaking, with the difference being the detection of the hydroxyapatite spectrum at 31.7&32.5°. The presence of these peaks serves as evidence of hydroxyapatite formation on the cement surfaces, confirming its bioactivity. Interestingly, the spectrum of samples NFC10 and NFC20 after soaking in solution is similar to that of NFC0, but the hydroxyapatite peaks appear more prominently, indicating that the addition of hydroxyapatite to the primary cement powders significantly enhances the cement’s bioactivity, which is suitable for dental purposes [47][48][49][50][51]. In order to facilitate comparisons, we’ll reference a comparable sequence of events in this discussion. These stages are depicted in an uncomplicated diagram included in Figure-10.

The functioning of the mechanism can be described in the following manner:

Once NFC comes into contact with SBF, a boundary forms between the cement and solution, leading to hydrolysis (stage 1) [52][53]. Ion exchanges commence between hydration products or C3S and C2S, with Ca2+ from the cement swapping with H+ from the aqueous solution to establish a solid-liquid interface. This interaction between Ca2+ ions and hydroxyl ions from water generates an alkaline environment (stage 2). By increasing the OH- concentration in the solution, cation exchange leads to the coating of calcium silicate particle surfaces with hydroxyl ions [54]. The process of hydrolysis produces the SiO44- grouping, aiding in the development of calcium silicate hydrate on cement surfaces in basic environments. This structure has no clear shape [55]. The structure of calcium silicate hydrate consists of a water-saturated gel formed by minuscule, porous particles that house silanol or Si-OH groups. Subsequently, in a basic environment, the Si-OH group within the calcium silicate hydrate releases a proton (H+). Through this process, a surface is generated that carries a negative charge due to the presence of a SiO- group (Stage 3) [56]: that carries a negative charge due to the presence of a SiO- group (Stage 3) [56]:


\equiv \mathrm{SiOH} + \mathrm{H_2O} \;\;\Leftrightarrow\;\; \equiv \mathrm{SiO^-} + \mathrm{H_3O^+}

(2)

The negative group formed attracts positive calcium ions released into the solution due to electrical forces, resulting in an increased concentration of positive ions on the cement’s surface [57]:


\equiv \mathrm{SiO^-} + \mathrm{Ca^{2+}} \;\;\Rightarrow\;\; \equiv \mathrm{SiO^-} \ldots \mathrm{Ca^{2+}}

(3)

It consists of two layers that possess opposing charges, enabling other materials to adhere to its surface. Alternatively, the SBF solution comprises PO43- that breaks apart in the following way: (stage4) [35]:


\mathrm{H_2O} + \mathrm{PO_4^{3-}} \;\;\Leftrightarrow\;\; \mathrm{HPO_4^{2-}} + \mathrm{OH^-}

(4)

The addition of calcium silicate to a phosphate-rich solution, referred to as SBF, that has disassembled HPO42- promotes interactions between HPO42- and Ca2+ on the surfaces of the cement. As a result of this process, apatite is formed unevenly (stage5) [58]:


\equiv \mathrm{SiO^-} + \mathrm{Ca^{2+}} + \mathrm{HPO_4^{2-}} \;\;\Rightarrow\;\; \equiv \mathrm{SiO^-} \ldots \mathrm{Ca^{2+}} \ldots \mathrm{HPO_4^{2-}}

(5)

Based on the SEM&EDAX and XRD findings of NCFH0, NFCH10, and NFCH20, it can be observed that the bioactivity of NFCH10 and NFCH20 surpasses that of NFCH0. This is attributed to the fact that the bioactivity of the degradable material is directly linked to the speed at which the ionic products are released from the corroding surface in a physiological solution [30]. NFCH20 and NFCH10 exhibit a higher quantity of hydroxyapatite nanoparticles in comparison to NFCH0. Upon immersion in the SBF solution, the hydroxyapatite nanoparticles migrate into the solution, leading to an increase in the concentration of calcium and phosphorus ions in the solution. The calcium, phosphate, and silicate ions released from the surface of the calcium silicate nanocomposite eventually react with the ions coming from SBF, resulting in the formation of insoluble salts such as hydroxyapatite on the surface of the cement. The elevation in the concentration of calcium and phosphorus ions accelerates the formation of hydroxyapatite on the sample surface, signifying an enhancement in bioactivity [59][60][61][62][63]. The heightened ionic activity of NCH20, in comparison to NCH10, led to a greater amount of apatite formation on the sample surfaces, which is attributed to the increased quantity of primary apatite in the microstructure.

## Conclusion

This study focused on assessing the effects of integrating hydroxyapatite nanoparticles (NHA) into NFC regarding the time required for it to set and its potential for supporting biological life. This aims to generate suitable filler material. Research into the various phases revealed that a compound known as apatite developed on samples that had been immersed in simulated body fluid (SBF) for a duration of 14 days. This was additionally assessed and corroborated through observation of the microstructure. This indicates that NFC is beneficial for health and safe for utilization. The presence of 10% and 20% primary apatite in the samples increased bioactivity and notably decreased sample cytotoxicity. The nanocomposite NFC/NHA exhibited positive biological properties and could potentially be a safe option for restorative treatment.

## Conflict of Interest

None.
